# A Pilot Study of Omalizumab in Eosinophilic Esophagitis

**DOI:** 10.1371/journal.pone.0113483

**Published:** 2015-03-19

**Authors:** Denise Loizou, Benjamin Enav, Edina Komlodi-Pasztor, Pamela Hider, Julie Kim-Chang, Laura Noonan, Tabitha Taber, Suhasini Kaushal, Renuka Limgala, Margaret Brown, Raavi Gupta, Nader Balba, Ozlem Goker-Alpan, Amer Khojah, Oral Alpan

**Affiliations:** 1 Section on Immunopathogenesis, Food Allergy and Eosinophilic Disorders Program, O&O ALPAN, LLC, Fairfax, VA, United States of America; 2 Amerimmune, Fairfax, VA, United States of America; 3 Pediatric Gastroenterology of Northern Virginia, Lorton, VA, United States of America; 4 Lysosomal Storage Disorders Section, O&O ALPAN, LLC, Fairfax, VA, United States of America; 5 Gastroenterology Associates of Northern Virginia, Fairfax, VA, United States of America; Jackson Laboratory, UNITED STATES

## Abstract

Eosinophilic disorders of the gastrointestinal tract are an emerging subset of immune pathologies within the spectrum of allergic inflammation. Eosinophilic Esophagitis (EoE), once considered a rare disease, is increasing in incidence, with a rate of over 1 in 10,000 in the US, for unknown reasons. The clinical management of EoE is challenging, thus there is an urgent need for understanding the etiology and pathophysiology of this eosinophilic disease to develop better therapeutic approaches. In this open label, single arm, unblinded study, we evaluated the effects of an anti-IgE treatment, omalizumab, on local inflammation in the esophagus and clinical correlates in patients with EoE. Omalizumab was administered for 12 weeks to 15 subjects with long standing EoE. There were no serious side effects from the treatment. Esophageal tissue inflammation was assessed both before and after therapy. After 3 months on omalizumab, although tissue Immunoglobulin E (IgE) levels were significantly reduced in all but two of the subjects, we found that full remission of EoE, which is defined as histologic and clinical improvement only in 33% of the patients. The decrease in tryptase-positive cells and eosinophils correlated significantly with the clinical outcome as measured by improvement in endoscopy and symptom scores, respectively. Omalizumab-induced remission of EoE was limited to subjects with low peripheral blood absolute eosinophil counts. These findings demonstrate that in a subset of EoE patients, IgE plays a role in the pathophysiology of the disease and that anti-IgE therapy with omalizumab may result in disease remission. Since this study is open label there is the potential for bias, hence the need for a larger double blind placebo controlled study. The data presented in this pilot study provides a foundation for proper patient selection to maximize clinical efficacy.

**Trial Registration:**

ClinicalTrials.gov NCT01040598

## Introduction

Eosinophilic esophagitis (EoE) is an allergic inflammation of the esophagus characterized by an eosinophilic infiltrate in the esophageal mucosa, hyperplasia of the basal layer and papillary lengthening despite acid blocker therapy with proton pump inhibitors [1,2]. The pathogenesis of EoE is not well understood, but the disease is thought to be due to an allergic reaction to ingested food [3]. As part of an allergic reaction, at least two different pathways that are not mutually exclusive can drive eosinophils into esophageal tissue. The first, which we call the “conventional pathway” suggests IL-13 influence upon esophageal epithelial cells to produce eotaxin, a chemokine that attracts eosinophils [4]. The second, which we will call the “alternative pathway” suggests an IgE driven disease process [5]. As can be seen in other allergic disorders, additional mediators of eosinophilic disease such as IL-5 and signaling through the surface receptor Chemoattractant Receptor-homologous molecule expressed on Th2 cells (CRTH2) also play a role, but it is unclear where they fall into the current understanding of this disease [6,7]. It is also possible for multiple pathways to play role in the induction of EoE. A good example for this is in patients undergoing oral food immunotherapy or sublingual immunotherapy for pollen allergy [8–11]. In such settings, the repeated administration of an allergen, which clearly induces IgE mediated inflammation can skew towards an eosinophilic response. This can be related either to dose or frequency of the orally administered antigen [10,11].

EoE is currently considered a public health problem reported in every continent except Africa. A recent study retrospectively examined 35,575,388 patient records from U.S. healthcare plan claims data. The case definition of EoE was any instance of the use of ICD-9 code 530.13 was used. The overall prevalence rate, standardized to the U.S. population, was 56.7/100,000. The prevalence was higher in men compared with women, and peaked in the 35-to-39-year age range, decreasing after age 45 [12].

Patients with EoE have an increased incidence of atopic disorders with increased IgE mediated food and inhalant sensitivities [13]. Use of either a targeted food allergen avoidance approach (based on allergy testing) or untargeted approach (based on food or environmental allergen avoidance) results in the resolution of eosinophilia in the gastrointestinal tract of approximately 50–70% of adult patients [14]. Compared to adults, children have a higher success rate in responding to food avoidance, ranging between 60–96% depending on the study design [3]. In one pediatric trial, the introduction of elemental formula, combined with strict food avoidance, resulted in clinical and histological disease remission in over 96% of the patients [15]. Although patients with EoE commonly go through in vivo (e.g. percutaneous or patch) or in vitro (e.g. ImmunoCap) testing in clinical practice, most do not show any positives to the foods to which lead to the accumulation of eosinophil in their esophagi. This indicates a discordance between currently available testing methods and clinical reactivity. Furthermore, EoE patients rarely become tolerant to their allergens, converse to current knowledge about conventional IgE-mediated allergies, such as milk, egg, and soy allergies, in which over 50% of cases result in remission [16–18].

In a recent study of a related but much more rare disease, eosinophilic gastroenteropathy, subjects who were treated with omalizumab [a humanized monoclonal antibody targeting the high-affinity receptor binding site on human immunoglobulin (Ig)E] in conjunction with standard therapies (such as dietary restriction), demonstrated reduced absolute eosinophil counts (AECs) in blood, as well as a decrease in eosinophil numbers both in gastric and duodenal mucosa but not reaching statistical significance. Esophageal eosinophil counts in these patients remained unchanged [19]. Peripheral blood basophil and dendritic cell FcRI expression and free IgE levels were significantly reduced.

Eosinophilic allergic disorders are very heterogeneous in their presentation as well as response to therapy [20–22]. Such heterogeneity has led to difficulties in designing clear therapeutic guidelines. For example, the clinical symptom scores in EoE do not necessarily correlate with the histological findings in the esophagus biopsy samples. Some patients may show a reduction in tissue eosinophil counts without clinical remission, while others undergoing clinical remission may have unchanged eosinophil counts in esophageal biopsies [23,24]. The lack of a biomarker predicting natural course or response to a particular treatment further complicates the management of this disease [25].

In this study, we sought to examine the effect of anti-IgE therapy on tissue eosinophil and mast cell counts, blood eosinophil counts, and their correlation to symptoms and endoscopy scores of patients with EoE.

## Methods

### Study oversight

The study protocol (Identifying Responders to Xolair (Omalizumab) Using Eosinophilic Esophagitis as a Disease Model, ClinicalTrials.gov Identifier: NCT01040598) was designed, written, edited, and the data were stored and analyzed, by employees of the sponsor, O&O ALPAN, LLC (S1 Protocol). The clinical investigators reviewed the protocol and collected the data. All authors reviewed and approved all drafts and made the decision to submit the manuscript for publication. All authors vouch for the accuracy and completeness of the reported data and for the fidelity of this report to the study protocol and statistical analysis plan. Ethics statement: The protocol was reviewed and approved by the Copernicus Group Institutional Review Board on February 10, 2009 and amendment to extend the age range to 75 on December 1, 2009. All participants provided written informed consent or assent. Study was registered with clincaltrials.gov on December 27, 2009 (NCT01040598).

### Study design

This study examined the effects of omalizumab on the allergic inflammation in the esophageal tissue of subjects with EoE. The unblinded, open-label, single center study was conducted from January 2009 through April 2011. Each subject’s omalizumab dose was calculated in mg/kg per international IgE units/ml (Table 1), as published in a similar study, which evaluated the use of omalizumab in eosinophilic gastrointestinal disease [19], similar to the dosage schedule used in allergic asthma [26]. Intention was to enroll a total of 24 patients, and divide patients into subgroups based on co-localization of IL-5 and IgE to tryptase positive mast cells. Study under enrolled and prevented us from making this correlation. We then proceeded to analyze the data by looking at overall response rate of the 15 subjects that met eligibility criteria.

**Table 1:** Study Subjects and Baseline Characteristics.

Subject Number	Disease Location	Presenting Symptom	Age (y/o)	Sex	Omalizumab Dose (mg/q/wks)	Disease duration (years)	Peripheral Blood AEC (cells/mcl)	IgE (IU/mL)	Esophagus Tissue Eos count/hpf*
1	m	Abdominal Pain, Dysphagia, Nausea	35	F	300 q4	10	100	109	15
2	d	Abdominal Pain, Vomiting, Dysphagia	16	F	150 q4	3	600	64	15
3	m	Abdominal Pain, Dysphagia	17	M	225 q4	4	314	301	50
4	m/d	Abdominal Pain, Dysphagia, Nausea	22	M	150 q 4	5	407	93	40
5	m/d	Dysphagia	71	F	1560 q4	12	255	47	30
6	m/d	Dysphagia, Heartburn	14	M	150 q4	3	252	41	40
7	m/d	Dysphagia, Impaction	15	M	375 q4	3	445	702	30
8	m/d	Abdominal Pain, Dysphagia	14	M	300 q4	4	228	482	25
9	m/d	Chest Pain, Throat Clearing	15	M	375 q4	4	680	619	50
10	m/d	Abdominal Pain, Dysphagia, Vomiting	14	F	300 q4	5	155	130	15
11	m/d	Cough, Dysphagia, Throat Clearing	12	M	300 q4	4	644	425	40
12	m/d	Dysphagia, Impaction	15	M	375 q4	7	607	530	30
13	m/d	Dysphagia	15	M	300 q4	3	154	253	30
14	m/d	Dysphagia, Food Impaction	13	M	300 q4	2	424	288	40
15	m/d	Chest pain, Gagging	18	M	150 q4	6	20	66	30

M: mid; d: distal, m/d: mid and distal.

*Represents the peak count either in distal or mid esophagus.

**Fig 1:**
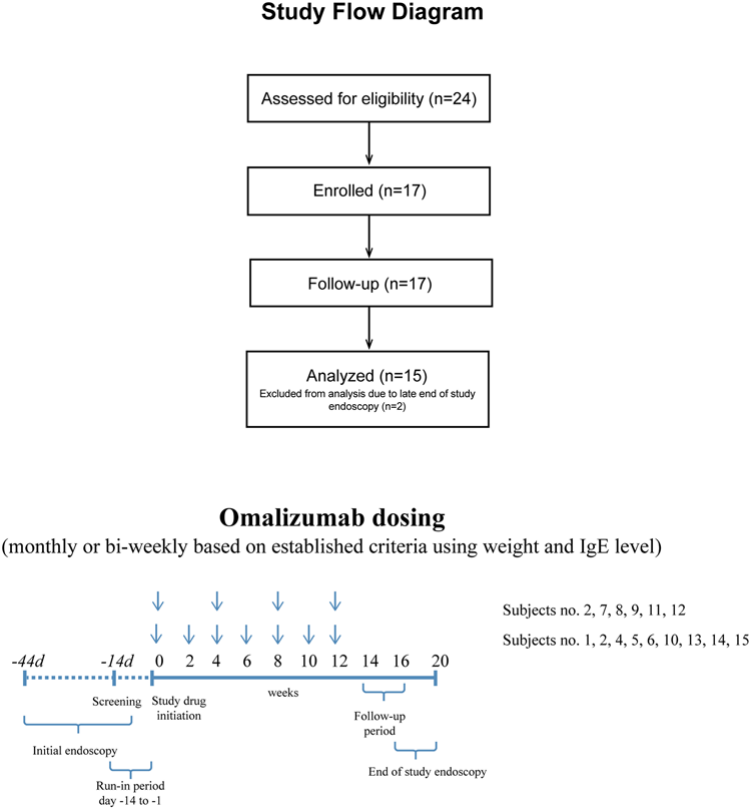
Study flow diagram and design. 24 subjects consented to participate in the study, 17 enrolled and completed all study visits. A total of 15 subjects who fully met inclusion and exclusion criteria, completed all study visits and drug administration, and underwent pre-and post-treatment endoscopic examinations were included in the data analysis. After a 2-week screening period, subjects received omalizumab subcutaneously every 2 weeks (6 patients) or 4 weeks (9 patients) for a total of 12 weeks. Safety and efficacy continued to be monitored during the follow-up period (weeks 12 through 16). The end of study endoscopy was performed between week 20 and 24.

Eligibility of the patients was established during the 2-week run-in period. During the 2-week pre-omalizumab baseline screening, subjects underwent esophagoduodenoscopy with biopsy and percutaneous skin testing using commercial allergens (Greer Laboratories, Lenoir, NC). All subjects enrolled into the study had IgE mediated allergy to at least one food allergen, based on skin prick testing. Subject medications and dietary restrictions were maintained without any changes throughout the study. The run-in period was followed by omalizumab injections subcutaneously every 2 or 4 weeks (depending on an established omalizumab dosing schedule for allergic asthma) for a total of 12 weeks. Subjects were observed for 2 hours after the first two doses and for 1 hour after subsequent doses. During the treatment period patients recorded their symptoms on survey cards. At study visits through week 12, assessments included: safety evaluation, blood testing, outcome questionnaires and study drug administration, if applicable. Safety and efficacy continued to be monitored during the follow-up period (weeks 12 through 16). Repeat endoscopy was performed between week 16–20. Fig. 1 outlines the study design. Subject medications and dietary restrictions were maintained consistently throughout the study.

### Subjects

Patients were recruited from the Food Allergy and Eosinophilic Disorders Program of O&O ALPAN, LLC. A total of 24 subjects consented to participate in the study and 17 completed all study visits and drug administration. Two subjects failed to obtain end of study endoscopies on time, hence were excluded from the data analysis. A total of 15 subjects who fully met inclusion and exclusion criteria, completed all study visits and drugs administration, and underwent pre-and post-treatment endoscopic examinations were included in the data analysis. All enrolled subjects tested negative for other potential causes of gastrointestinal eosinophilia, including helminth infection, drug reaction and hypereosinophilic disease. Crohn’s disease was ruled out by lack of pathologic findings (ulcerations, granulomata, or crypt architectural distortion) and clinical features (fistula, abdominal mass, and surgical obstructive disease) consistent with the disease. Table 1 shows the baseline clinical findings of enrolled subjects.

The following inclusion criteria was used for selection of the subjects: age 12 to 75 years, evidence of atopy by skin or serologic testing, total serum IgE level between 30–700 IU/mL, eosinophilia of the esophagus >15 eosinophils/hpf despite the use of a proton pump inhibitor (PPI) for at least two months with repeat endoscopic evidence of esophageal eosinophilia before entering the study, failure to treat the symptoms with either food avoidance or oral steroids and one active symptom of disease (epigastric pain, vomiting, dysphagia, or heartburn) at least two days of the week.

Study subjects also had to demonstrate that they did not meet the following exclusion criteria: physician’s diagnosis of immunodeficiency, IgE level of greater than 700 IU/ml, medical history of eosinophilic gastroenteropathy or gastrointestinal reflux disease, peripheral eosinophil counts greater than 1500/μl, known sensitivity to study drug or class of study drug, use of any other investigational agent in the last 30 days, use of systemic or inhaled steroids within the past month, history of malignancy; use of chronic immunosuppressive therapy (including but not limited to cyclosporine and methotrexate) or use of omalizumab within the 12 months prior to screening.

The study inclusion and exclusion criteria were designed to select a relatively homogenous population in order to enhance interpretation of the clinical and histopathology findings from patients before and after omalizumab therapy.

### H&E and immunohistochemistry

Endoscopic biopsy specimens were taken from the distal third and middle third of the esophagus, gastric antrum or the body, as well as the duodenum. A total of three biopsy specimens were taken from each level, namely the mid and distal esophagus, and fixed in formalin. A pathologist and an independent investigator reviewed hematoxylin and eosin stained slides. The number of eosinophils per high power field (HPF) was measured in the area of highest severity. Peak eosinophil count from the mid and distal esophagus was used for data analysis. Quantitation of mast cells (tryptase positive cells) and IgE positive cells was performed on immunostained sections with a Leitz microscope (Laborlux S microscope, E. Leitz, Wetzlar, Germany) at 40-x HPF magnification. The number of cells stained positive were counted in eight contiguous non-overlapping fields and expressed as mast cells per hpf (MC/hpf) and IgE-positive cells per HPF.

For immunohistochemistry, formalin fixed paraffin-embedded samples were cut in 5-μm sections. Endogenous peroxidase activity was blocked with 0.2% hydrogen peroxidase solution and non-specific labeling was blocked in serum blocking solution. Sections were incubated in complete medium for 1 hour at room temperature with rabbit anti-human tryptase (1:400) and goat anti-human IgE (1:400) monoclonal antibodies, overnight at 4°C. As a negative control the primary antibody was omitted and replaced with phosphate-buffered saline. The reaction was revealed by the avidin-biotin complex peroxidase method (ABC Elite kit, Vector, Burlingame, CA, USA) followed by staining with the peroxidase substrate 3,3′-diaminobenzidine tetrachloride (DAB; Sigma, Deisenhofen, Germany). The slides were counterstained with 50% hematoxylin.

### Esophagoduodenoscopy

Endoscopy was performed on all subjects and the following findings were documented: fixed rings (trachealization); exudates (plaques or white spots); furrows (vertical lines and longitudinal furrows); edema (mucosal pallor) and stricture. To be consistent, endoscopic findings were graded for severity by the principal investigator based on the endoscopy reports. The grading system were as follows: Grade 0–3 for rings, Grade 0–2 for exudates, furrows, edema and stricture [27].

### Symptoms score

To measure Eosinophil-associated GastroIntestinal Disorder (EGID) symptoms, we used a modified version of the well-accepted Crohn’s Disease Activity Index (CDAI). Although this scoring system is not currently validated in EoE, it has successfully been utilized in a prior study of patients receiving omalizumab for EGIDs. Patients were provided symptom survey cards to rate their symptoms on a weekly basis. Subjects rated vomiting, nausea, bloating, early satiety, dysphagia, abdominal pain and general well being on a scale from 0 to 3, and documented the number of events every week. Symptom score was calculated as the sum of these scores [19].

### Cell preparation and flow cytometry

For flow cytometric analysis, blood was collected in BD Vacutainer containing ethylenediaminetetracetic acid (EDTA) to prevent coagulation. The samples were processed within 6 hours of collection to reduce activation of eosinophils after collection. The samples were incubated for 30 minutes at 4°C with Anti-CD69 mAb bound to fluorescein isothiocyanate (FN50, mIgG1, BD Pharmigen), Anti- FcR1 mAb bound to allophycocyanin (AER-37, mIgG2, eBioscience), Anti-CD9 mAb bound to R-phycoerythrin (eBioSN4, mIgG1, eBiosciences), Anti-CD16 mAb bound to peridinin chlorophyll protein (3G8, mIgG1, Invitrogen). Samples were lysed using FACS Lysing Solution (BD Biosciences) following manufacturer’s instructions. Cells were washed using PBS solution and suspended in 1% formaldehyde solution and analyzed on an AcuriC6 Flow Cytometer (BD Biosciences).

### Statistical analysis

Statistical analysis was done using the Statistical Package for the Social Sciences (SPSS) version 21. We decided to use paired samples *t*-tests consisting of a group of units that has been tested twice (a "repeated measures" *t*-test). The subjects were tested prior to treatment with omalizumab, and the same subjects were tested again after treatment. The paired t-test was used to compare between variables in pre- and post-intervention groups. A non-parametric statistical hypothesis test, Wilcoxon signed-rank test, was also used to compare the correlations between changes in pre- and post-intervention data points. P values less than 0.05 were considered to be statistically significant. The graphs were produced using EazyDraw version 5.3.1. The analysis performed in this study is as per protocol and does not indicate intent-to-treat.

## Results and Discussion

### Impact of anti-IgE therapy on the clinical disease in EoE

**Fig 2:**
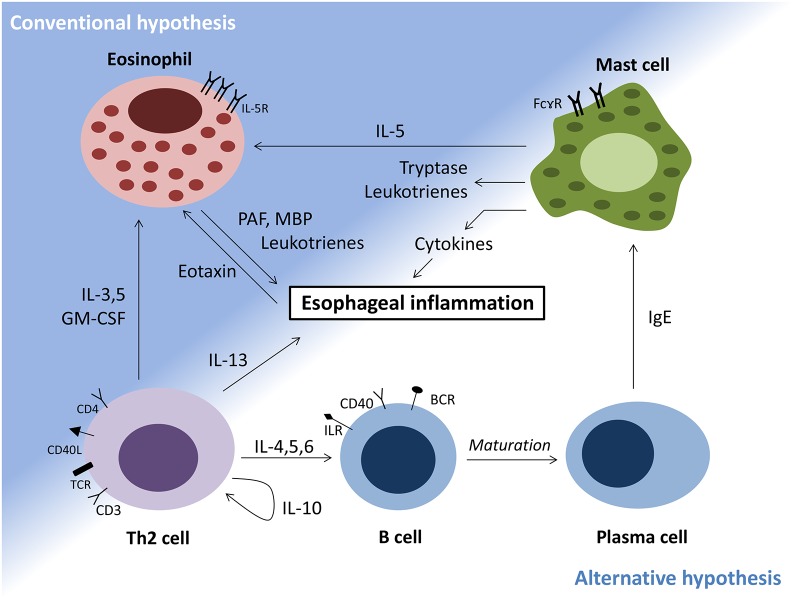
Mechanisms of eosinophilic esophagitis. Conventional hypothesis: esophageal epithelial cells mediate eosinophil influx into esophagus. Alternative hypothesis: IgE mediated secretion of eosinophilic factors from mast cells.

To dissect the role of IgE in the pathology of EoE, we designed a clinical study in which patients with active EoE received omalizumab for a total of 12 weeks. We hypothesized that IgE has a role in the mechanism of EoE and that blocking IgE would improve disease symptoms and reduce allergic inflammation, as measured by a decrease in esophageal tissue eosinophilia. Since T cell derived chemokines play a role in eosinophilic attraction to tissues as part of the “conventional pathway”, IgE blockade would only resolve the mechanism as described in the “alternative pathway” in which IL-4 driven IgE production is the main driving force for the disease (Fig. 2). Clinical and immunological parameters were assessed before and 12 weeks after omalizumab therapy. The duration of omalizumab therapy of 3 months was chosen based on the current clinical practice of time to assess disease activity after the introduction of a new treatment modality.

Characteristics of the patients are shown in Table 1. A total of 24 subjects were approached and enrolled, including 17 who completed the study and only 15 were analyzed who completed all study related procedures. Eleven of the 15 patients were male, as anticipated at study onset due to previous observations that EoE affects predominantly males [2].

The average age at enrollment was 20.4 years (ranging from 12 to 71 years of age) and the median of age was 14 years. The most frequently observed symptoms included dysphagia (13 of the 15 patients), abdominal pain (6 of the 15 patients), and nausea and/or vomiting (4 of the 15 patients). The average time for disease duration was 5 years (ranging from 2 to 12 years), and the median disease duration was 4 years. The average pre-treatment eosinophil count per hpf in the esophageal tissue was 30.2 (ranging from15 to 50), and the median was 30 eosinophils per hpf.

A total of 78 doses of omalizumab were administered with no observed severe adverse events. Of the 78 doses, 60 doses were administered to pediatric age group (<18 years old). Laboratory studies done for the assessment of safety, which included a comprehensive metabolic panel, complete blood count, serum quantitative immunoglobulin titers, and lymphocyte subsets, showed no significant deviations form normal ranges (data not shown). There were no incidences of anaphylaxis, serum sickness, cardiovascular events or malignancy during the course of the study. The study safety data confirmed previous safety data published on the use of omalizumab in eosinophilic gastrointestinal disorders [19], chronic idiopathic urticaria (CIU) [28], and asthma [26,29].

**Fig 3:**
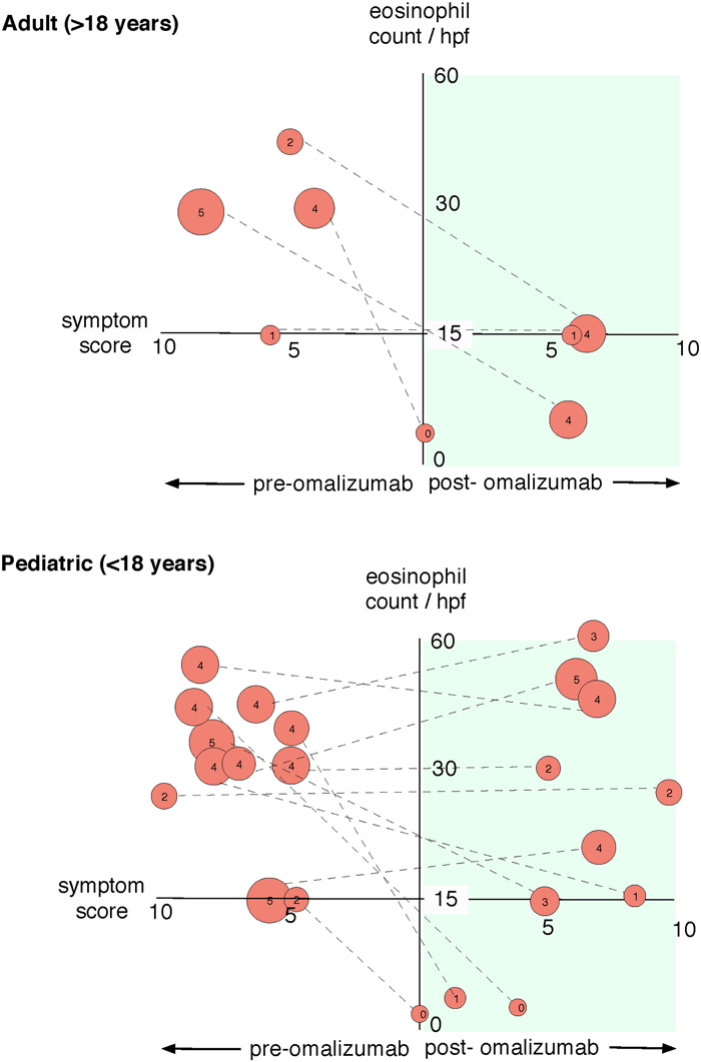
Effect of omalizumab on esophageal eosinophil counts, symptom scores and overall endoscopic score. The x-axis represents symptoms scores, the y-axis eosinophil counts, and right side of the graph represents post therapy with omalizumab. Individual patient data are shown as circles connected with a dashed line to allow comparison between pretreatment and post treatment. The size of the circle represents endoscopy score (also shown as a number inside the circle).

We first analyzed clinical symptom data in correlation with the endoscopy findings and tissue eosinophil counts (Fig. 3). Eosinophil-associated gastrointestinal disorders (EGID) symptom scores improved at the end of study (during weeks 15–16) in 7 out of 15 subjects with an overall change in total combined symptoms score from 6.6 to 5.4 which was found to be statistically significant (p = 0.018) (Tables 2 and 3). In five subjects with EoE, full disease remission was accomplished with the use of omalizumab (shown as red connecting lines in Fig. 3). Full disease remission is defined as histological (< 15 eosinophils/hpf in the esophagus biopsies) and endoscopic resolution of disease. This translates to 33% overall full remission rate with omalizumab. We did not observe any symptomatic improvement in any of the subjects who did not show histological improvement. There were no statistically significant correlations of changes in symptoms scores before and after omalizumab to changes in blood AECs (Table 4). When we compared histological changes in eosinophil numbers to symptom score changes before and after omalizumab therapy, there was a statistically significant correlation (p = 0.0276, Fig. 3 & Table 4).

**Table 2:** Summary of the statistics of the comparison of pre and post omalizumab data points: Paired T-Test.

	Pre-Omalizumab	Post-Omalizumab	95% Confidence Interval of the Difference		P Value
	Mean	Mean	Lower	Higher	
Peripheral blood AEC	359.0	359.7	-123.68804	122.35471	0.991
Tissue Eosinophil	31.0	19.6	0.97478	21.82522	0.034
Tissue Mast Cell	23.7	14.2	3.72950	15.33717	0.003
Tissue IgE	16.5	3.5	5.87732	20.12268	0.002
Symptom Score	6.6	5.4	0.23627	2.16373	0.018
Endoscopy Finding	3.6	2.2	0.37888	2.42112	0.011

**Table 3:** Summary of the statistics of the comparison of pre and post omalizumab data points: Wilcoxon Signed-Rank Test (2-tailed).

	Z-Value	W-Value	Standard Deviation (W)	P Value
Peripheral blood AEC	-0.0568	59	17.61	0.952
Tissue Eosinophil	-2.0396	13	12.75	0.041
Tissue Mast Cell	-2.7011	1	9.81	0.006
Tissue IgE	-3.2958	0	15.93	0.001
Symptom Score	-2.2934	5	9.81	0.03
Endoscopy Finding	-2.3534	9	12.75	0.018

**Table 4:** Correlation of the changes in histology, endoscopy, symptom scores and blood eosinophils before and after omalizumab.

	Changes in MC	Changes in Eos	Symptom Changes	Endoscopic Changes	AEC Change	Furrowing Change
**Changes in MC**	-	0.1292	0.0507	0.0177	0.5451	0.0373
**Changes in Eos**	0.1292	-	0.0276	0.0619	0.5814	0.0188
**Symptom Changes**	0.0507	0.0276	-	0.0422	0.6450	0.0519
**Endoscopic Changes**	0.0177	0.0619	0.0422	-	0.6513	<0.0001
**AEC Change**	0.5451	0.5814	0.6450	0.5451	-	0.7939
**Furrowing Change**	0.0373	0.0188	0.0519	<0.0001	0.7939	-

When we stratified patients based on age (pediatric < 18 years of age, adult ≥ 18 years of age), only one of four adult subjects demonstrated full remission (Fig. 3). In pediatric patients, histological remission was evident in four out of eleven subjects. The total number of subjects in each age category as identified above was not sufficient for a sub-group statistical analysis. Neither subject age nor pre-treatment esophageal eosinophil counts correlated with response (or induction of remission) to omalizumab.

The full remission rate we see in our study is in contrast to what was observed in the use of two other biologicals that target IL-5 to treat EoE (mepolizumab [30–32] and reslizumab [33]), which resulted in a robust anti-eosinophilic effect but no statistically significant improvement in disease remission despite promising results in the pilot study of mepolizumab in four patients [30].

Similarly in a study by Straumann et al, where patients with active EoE either resistant to or dependent on steroids were selected and treated for 8 weeks in a randomized, double blind, placebo controlled trial with a CRTH2 antagonist (6). Only a modest but significant anti-eosinophilic effect was observed. Patient heterogeneity, differences in age, sex, histological and morphological severity, duration and the extent (beyond the esophagus) of the disease and its responsiveness to steroid therapy will all have influence on the outcome of these trials. These kinds of variations make it difficult to make a clear comparison between these independent trials.

In a recently published placebo controlled study, omalizumab did not show any improvement in esophageal eosinophil counts or symptoms in patients with EoE [34]. This study enrolled a total of 30 subjects with EoE, of which 16 were dosed with omalizumab for a duration of 4 months followed by repeat endoscopy to evaluate efficacy. Compared to our trial, this study had a placebo control arm, had a slightly older patient population (20.4 years versus 32 years) and similar serum total IgE levels (276 IU/ml versus 362 IU/ml). Although disease remission was not observed, lack of reporting peripheral blood eosinophil counts at study onset, especially given our findings of positive response to omalizumab in patients with low peripheral blood AEC at study initiation, as well as atopic status of individuals (all subjects in our study have evidence of atopy based on skin testing) limits comparison of both studies and may explain the differences in outcome. Similarly, the results from the two other published cases demonstrating the failure of omalizumab to induce remission of EoE is also difficult to extrapolate to our study and to that of Fang et al due to the age of the first subject being less then 8 years and in the second case the IgE level being out of range, making dose calculation difficult in both cases due to lack of any established guidelines [34,35].

### Omalizumab-treated EoE patients display favorable improvement in esophageal morphology on endoscopy

**Fig 4:**
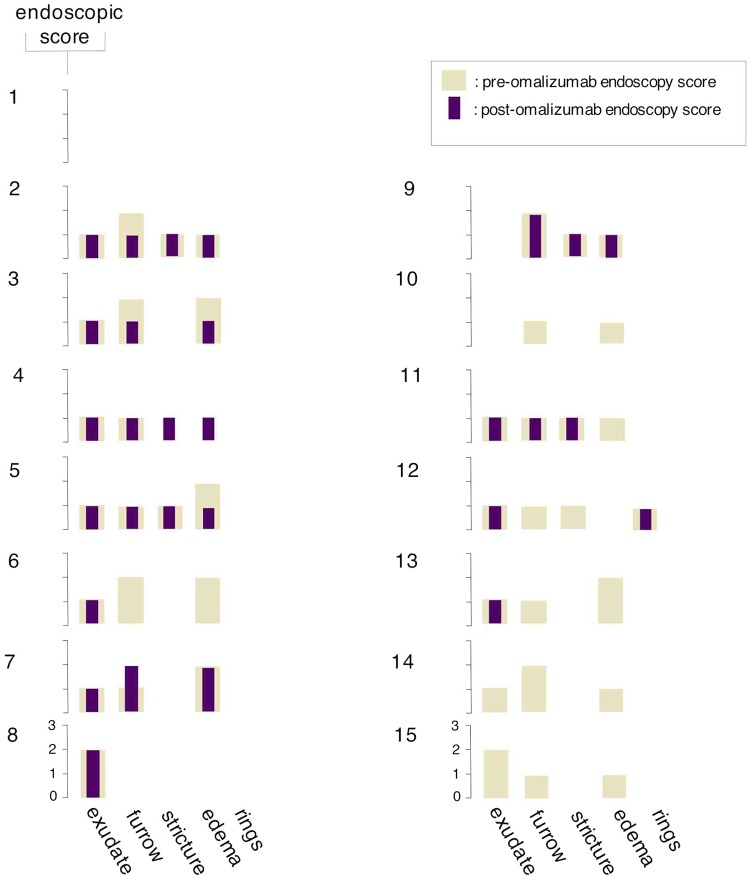
Endoscopic scoring of individual patients before and after therapy with omalizumab. The y-axis represents symptoms scores; the x-axis represents endoscopic findings. The pale bar represent endoscopic score before therapy with omalizumab, and the purple bar represents endoscopic score 3 months after therapy with omalizumab. Numbers to the upper left of each graph shows individual patients. Endoscopic scores are graded as: 0–2 for exudates, 0–2 for furrows, 0–1 for stricture, 0–2 for edema and 0–3 for rings.

Patients receiving omalizumab therapy had an overall statistically significant improvement in endoscopic findings based on a reduction of endoscopic scores of disease activity from 6.6 to 5.4 (p = 0.18, Table 2). Changes in esophageal mast cells, but not eosinophils showed a statistically significant correlation with improvement in endoscopy scores (Tables 3 and 4). This finding suggests limited value of correlating endoscopic and histological findings. Of the five endoscopic findings characteristic of EoE (exudates, furrowing, edema, rings, and strictures), the finding that correlated with the changes in histology (both mast cell and eosinophils in tissue) was in furrowing (Table 4). When we compare the correlation between changes in total endoscopic scores with symptom score changes before and after omalizumab, we see a statistically significant correlation (p = 0.042, Fig. 4 & Table 4). It is possible that the furrowing is an IgE mediated process, and the lack of effect of omalizumab on other findings of EoE may lead to the lack of a statistically significant correlation between symptoms scores and endoscopic scores. The decrease in tissue eosinophil counts correlated with the decrease in symptom scores (p = 0.0276) and furrowing observed in endoscopy (p = 0.0188) but not overall total endoscopy scores (Table 3). Only one of the subjects demonstrated trachealization on endoscopy pre-omalizumab. In contrast to other endoscopic findings of EoE which can heal within a short time after initiation of therapy, trachealization has been described to be the most refractory finding to steroid therapy, which can take up to six months to heal [36]. In agreement with the literature, our subject with the findings of trachealization was also a poor responder to omalizumab.

### Omalizumab reduces esophageal tissue IgE levels

**Fig 5:**
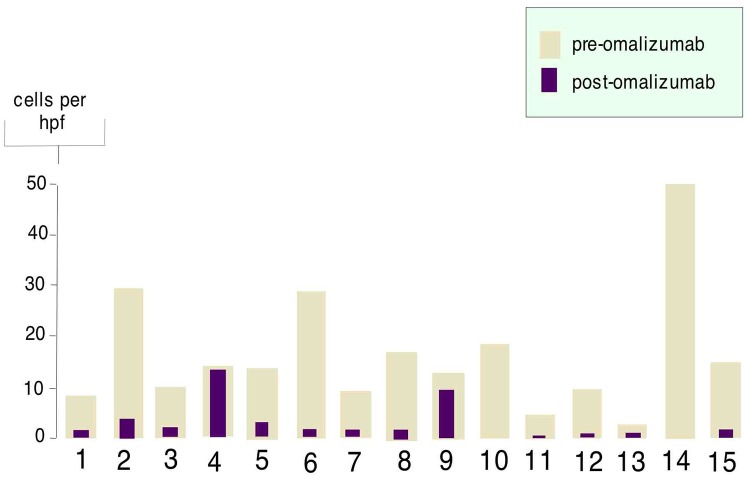
Effect of omalizumab on tissue IgE. Immunohistochemical staining for IgE was performed at baseline and 3 months into omalizumab therapy. Positive stain is shown in per high power field.

There was a reduction in esophageal tissue IgE staining after completion of omalizumab treatment (p = 0.002) from 16.5 spots/hpf to 3.5 spots/hpf (Fig. 5 & Tables 2 and 3). Similar findings in tissue IgE staining have been reported in other clinical studies of omalizumab, including in asthmatic airways [29] and in nasal biopsy specimens from allergic rhinitis patients [37], but in lesser statistical significance, suggesting possible tissue specificity of omalizumab. The primary antibody we used to detect tissue IgE recognizes an epitope separate from the binding side of omalizumab; hence the post-treatment reduction in the IgE levels is not due to competitive inhibition between the primary antibody and omalizumab.

### Correlation between esophageal eosinophils and mast cells in response to omalizumab therapy

**Fig 6:**
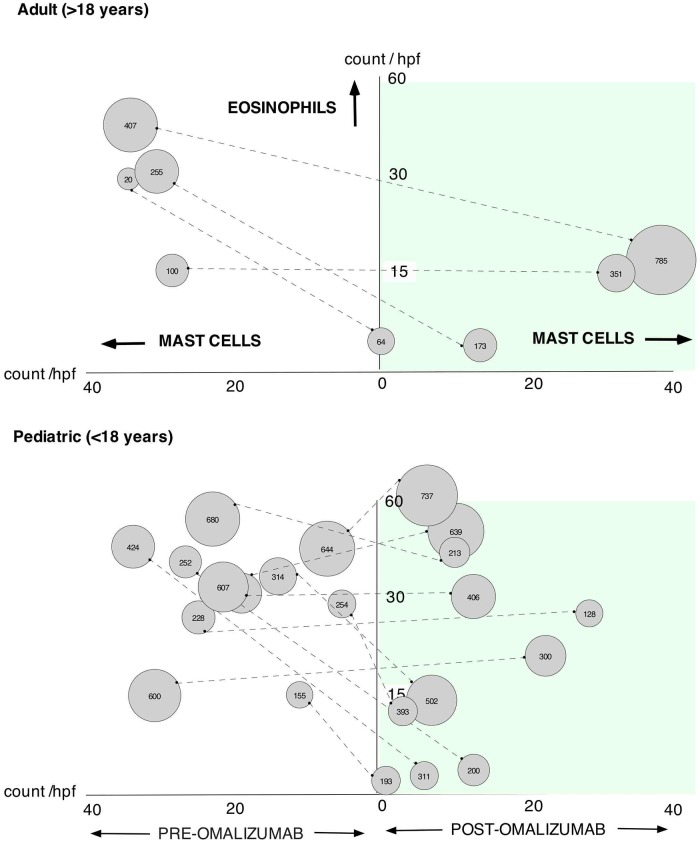
Relationship between mast cells and eosinophils in the tissue and their comparison to blood eosinophil counts. The x-axis represents mast cell count per high power field; the y-axis represents eosinophil counts per high power field. Left side of the graph is data before omalizumab therapy and right side of the graph after therapy. Individual patient data are shown as circles connected with a dashed line. The size of the circle represents peripheral blood absolute eosinophil count (also shown as a number inside the circle).

The correlation between blood and tissue eosinophils as well as tissue mast cells before and after omalizumab therapy is shown in Fig. 6. Mast cells were identified by tryptase staining using immunohistochemistry staining before and after treatment with omalizumab. Mast cells have been identified in patients with EoE as a part of the pathology along with other inflammatory cells [22]. There was a statistically significant reduction in esophageal mast cell counts per hpf after treatment with omalizumab (p = 0.006, Fig. 6 and Table 2), but this decrease did not show statistically significant correlation with a reduction in esophageal tissue eosinophil counts as well as blood AECs (Fig. 6, Tables 3 and 4). After omalizumab treatment 2 out of 15 subjects demonstrated a decrease in esophageal tissue mast cells despite persisting tissue eosinophilia and an additional 2 out of 15 patient had no change or increase in mast cell counts despite decrease in tissue eosinophil numbers after treatment with omalizumab (Fig. 6). In five subjects, the reduction in mast cells post-omalizumab showed a statistically significant correlation with changes in endoscopic score (p = 0.0177, Fig. 4 and Table 4). This is an unexpected finding since current understanding and expectations in the clinical management of EoE suggests eosinophil counts to correlate with visual improvements which we did not find to be statistically significant (p = 0.0619, Table 4). Larger prospective studies are needed to further evaluate this correlation.

Reduction in the esophageal tissue mast cell counts did not consistently accompany reduction in tissue eosinophil counts in every subject, therefore, an interdependency does not seem to exist for these two cell types within the esophageal tissue. In patients with clinical, histological and endoscopic remission, both eosinophil and mast cell counts showed a very dramatic decline. These results are in agreement with a recently published mechanism that shows a correlation between eosinophil and mast cell reduction in esophageal tissue after therapy with anti-IL-5, where it was proposed that the concomitant reduction of eosinophil derived IL-9 could account for the reduction of esophageal mast cells in these patients [38]. It is of note that in a previous study, which failed to show efficacy of omalizumab in EoE, a similar reduction in mast cells were observed [34].

Omalizumab-induced reduction in mast cells at the tissue level also raises the possibility of the same mechanistic action that leads to the efficacy of omalizumab in subjects with chronic idiopathic urticaria (CIU) [28], a disease in which mast cells play an important role. Although CIU does not present with dermal mastocytosis, omalizumab may alter mast cell activation thereby improving the clinical symptoms of CIU. In a small study of three patients, omalizumab recipients, but not control subjects, demonstrated reductions in Fc epsilon RI alpha immunoreactivity at days 70 and 196 in parallel with reductions in the acute wheal response to allergen challenge [39]. The effect of omalizumab alteration of mast cell activation warrants further investigation of this pathway’s role in CIU.

**Fig 7:**
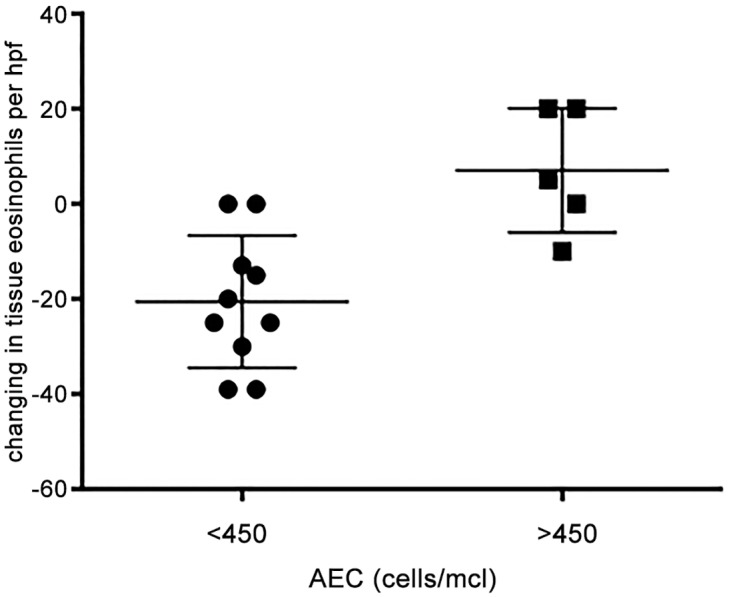
Change in tissue eosinophil counts/hpf in response to omalizumab therapy in patients with low and high peripheral blood absolute eosinophil counts. Patients were classified as having high (>450 cell/mcl) and low (<450 cells/mcl) peripheral blood eosinophil counts and the tissue eosinophil counts/hpf before and after omalizumab were compared using t-test (p = 0.0027).

**Fig 8:**
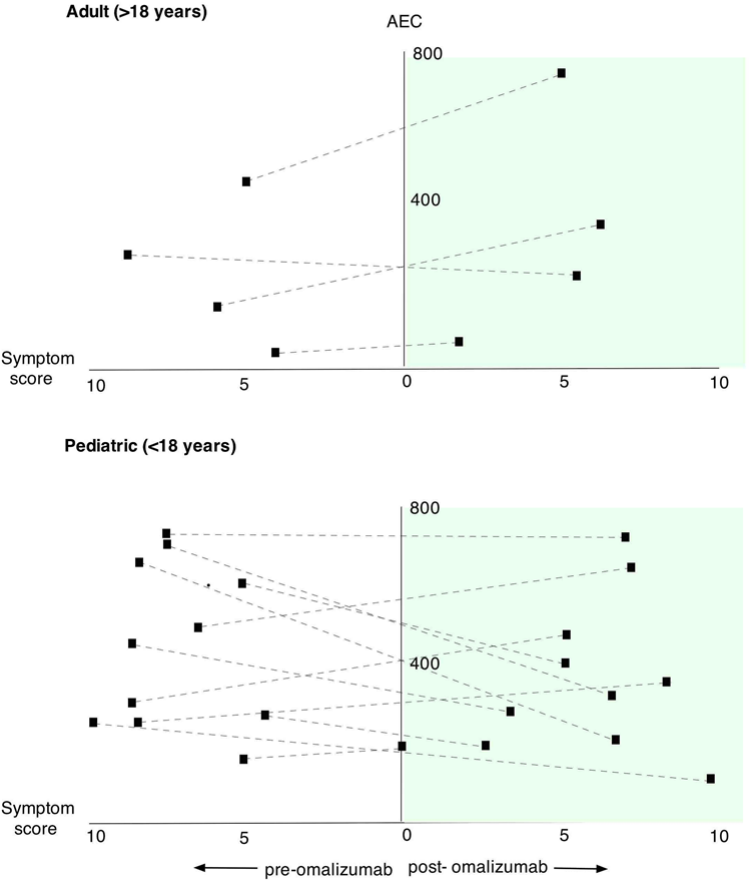
Comparison of blood AEC to symptom score. The x-axis represents symptoms scores before (left) and after (right) omalizumab, the y-axis represents AEC in peripheral blood.

Overall, changes in tissue eosinophil counts did not correlate with any changes in blood AECs (p = 0.5814, Table 3). When patients were stratified based on AEC count at baseline, there was a statistically significant correlation in patients with AEC < 450 (low-Eos) and histological response to omalizumab (p = 0.02). (Tables 2 and 3 and Fig. 7). Due to unequal number of subjects in high and low-eos groups, we were not able to perform a Wilcoxon signed-rank test, but compared the two groups by students t-test. When we compared the change in clinical symptom scores to changes in AEC, we found no correlation (Fig. 8, Table 4). In a recent study which included atopic asthmatics with elevated serum total IgE levels, high peripheral blood AEC was found to be a potential biomarker for assessing successful omalizumab treatment effects, which is in contrast to what we see in eosinophilic esophagitis [40]. In a study of omalizumab in patients with EGIDs [19], almost all patients had elevated blood eosinophil counts (> 500 per mm^3^at baseline) which may explain why histological improvements were not accompanied by clinical remission of disease. We see this contrast between histological responses to omalizumab in subjects with low versus high AEC (Fig. 7).

Our data indicates that IgE-mediated processes contribute to the generation of the eosinophilic inflammation in EoE, and that anti-IgE therapy might be effective in a subset of patients. Non-IgE responses, however, operate in an overwhelming majority of EoE patients. Larger, multi-center studies are needed to have an accurate estimate of the response rate to omalizumab, since smaller, single center studies, as reported here, may have patient selection bias. The magnitude of symptom improvement and reduction in tissue eosinophilia were variable between subjects, suggesting heterogeneity in the disease mechanism. Since all subjects were dosed based on body weight and IgE level, these findings are not strictly attributable to more effective IgE inhibition. Due to the heterogeneous nature of this disease, further studies are needed to identify clinical or histological biomarkers that would stratify patients into subgroups based on therapeutic needs. Patients enrolled in this trial were not steroid resistant, steroid dependent or required multiple dilatation procedures within the past year, which are characteristics of severe EoE.

In this open-label study subjects’ diets and medications were held constant, with omalizumab as the only variable introduced. Sources of error for this study include our inability to determine the strength of a “placebo effect” since there was not a placebo arm. Another complication in the interpretation of EoE results in any protocol is the variable nature of the disease activity within individual patients, in that some patients will spontaneously recover. Additionally, although we found no correlation between AEC and aeroallergen season (data not shown), it is possible that some subjects’ symptom improvement may have been influenced by changes in environmental allergen levels. Therefore, it may not be possible to attribute symptom improvement to the study drug alone.

EGIDs represent a spectrum of diseases that are increasing in incidence and lack safe and effective treatments. In our experience, food elimination strategies based on significant positives on skin prick testing combined with food patch testing only lead to histological resolution of disease in approximately 40% of patients (data not published), which is lower then the data published in the literature [3,15]. Even though non-targeted food elimination strategies (e.g. taking the top six foods such as milk, soy, egg, nuts/peanuts, wheat, and seafood out of the patients diet) has demonstrated favorable outcomes in a vast majority of patients [14,23]; given that EoE is a chronic disease, we find such diets to be almost impossible to maintain when offered to patients without an end date in place [1]. The lack of standardized diagnostic and treatment modalities and the fact that non-IgE mechanisms possibly play a role in over 50% of the patients in EoE dictates the need for specialized expertise to manage such patients. Specialized teams trained in managing EoE should include: immunologists, registered dietitians, feeding therapists, occupational therapists, and gastroenterologists. These teams are essential for identifying and managing patients with EoE. Furthermore, initiatives led by several national (e.g. Lysosomal & Rare Disorders Research and Treatment Center [*www*.*ldrtc*.*org*] and American Partnership For Eosinophilic Disorders [*www*.*apfed*.*org*]) and local (e.g. Washington Area Eosinophilic Connection [*www*.*washingtoneos*.*org*]) non-profit patient organizations play a tremendous role in taking the necessary steps to move the field forward to better understand EGID pathogenesis as well as improving diagnostics and therapy of this disorder [41].

At the present time, the only gold standard diagnostic method for identifying eosinophilic esophagitis is endoscopy with biopsy. This creates certain barriers to both diagnosis and disease management. Many adult patients as well as parents of pediatric patients of EoE are hesitant to have multiple repeated procedures that involve anesthesia. The development of non-invasive diagnostics for eosinophilic esophagitis is vital for improved treatment compliance and better patient outcomes. Research should be focused on progressing techniques like the string test [42] and also continuing to look for other biomarkers [25,43].

There is a significant body of evidence on the role of IgE in patients with EoE. Total IgE levels are increased (>114 kU/L) in 50% to 60% of patients with EoE. Higher total IgE levels are reported in allergen-sensitized versus non-sensitized patients with EoE [44]. At the present time, however, there are inadequate data to support the utility of measuring the total IgE level as a surrogate disease indicator of histologic inflammation in patients with EoE. The presence of allergic rhinitis, sensitization to aeroallergens, or both, ranges from 24% to 78% in adult patients and 42% to 93% in children with EoE. Sensitization to pollens that cross-react with plant-derived food allergens, such as in oral allergy syndrome, might provide a link between pollen sensitization and subsequent food ingestion in triggering EoE, although studies to address this possibility are currently lacking [44]. When IgE against food allergens was evaluated, among adult patients, 50% had positive results to at least 1 food, the most common being peanut (38%), egg (27%), and soy (23%). In children these rates are higher than generally reported from the adult studies. Although studies support a high rate of sensitization to foods in patients with EoE, and a subset of patients with EoE might have acute allergic reactions to foods, warranting evaluation for IgE-mediated food allergies, there are limited data addressing the diagnostic value of skin prick tests for identifying foods that might directly contribute to EoE [44].

**Fig 9:**
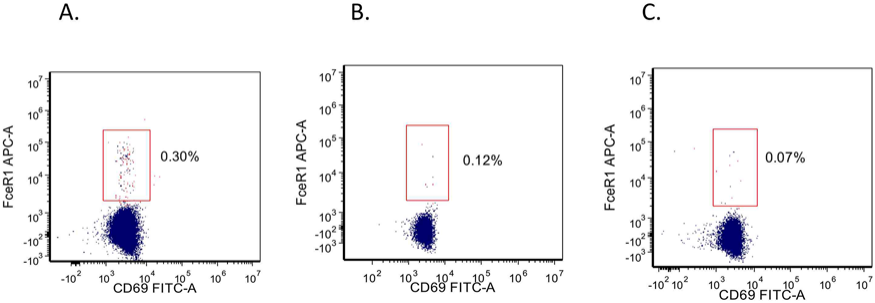
Eosinophil FcR1 expression in EoE. Eosinophils were identified by a combination of FSC/SSA and expression of CD9 and CD16 as CD9^+^CD16^-^ cells. Evaluation of FcεR1 and CD69 were compared versus IgG1 as an isotype control.

It is also possible that the high affinity IgE receptor (FcRI) on eosinophils may play a role in the effects of omalizumab in patients with EoE. FcRI is present on the cell surface of mast cells, basophils and on antigen presenting cells (APCs) such as dendritic cells (DCs) and monocytes [19]. In some atopic conditions, the surface expression of FcRI is increased [19]. Depending on the strength of the stimulus through the FcRI a distinct cytokine and chemokine production in mast cells has been observed [45]. Simultaneous to the omalizumab-induced reduction in the level of circulating free IgE, a corresponding decrease in surface FcRI expression by peripheral blood basophils as well as a rapid decrease in surface FcRI expression by both the pDC1 and pDC2 subsets are seen [19,46]. When we looked at this receptor in a separate cohort of EoE patients, we detected a wide spectrum of surface expression profile of FcRI on eosinophils that does not correlate with circulating eosinophil cell numbers or serum IgE levels (Fig. 9). Hence this heterogeneity may explain the differences in response to various therapies that one sees either within or between studies of EoE. The correlation between peripheral blood eosinophil counts, their activation status and response to omalizumab in EoE merits further investigation of its use as a biomarker to guide anti-IgE therapy in patients with EoE.

EGIDs represent a spectrum of diseases increasing in incidence, which lack safe and effective treatments. Progress in understanding EGID pathogenesis is needed to improve therapy. Our results demonstrate that omalizumab is effective in reducing esophageal tissue eosinophilia in EoE, a type of EGID, which suggests an IgE-mediated process plays a pivotal role in the pathophysiology of a subgroup of EGID patients. Therefore, anti-IgE therapy may be an effective treatment to pursue for EGIDs.

Even though we have shown a correlation between mast cells, eosinophils and clinical/endoscopy scores, further studies are needed to elucidate underlying mechanisms of individual variability and response to immunomodulator therapies. In order to compensate for individual variability, we recommend considering crossover studies, in which the patient acts as their own control, and in which anti-IgE as well as other drugs of interest, such as anti-IL-13, are administered sequentially following adequate washout periods. Universal endpoints, such as disease flare-ups requiring steroid therapy and dilatation, should be agreed upon to help reduce inter-study variability. There is also a need to understand disease variability over time to enable the rational design of studies that are longer in duration (e.g. 2–3 years). This is especially important from a therapeutic standpoint in understanding whether fluctuations of disease activity happen over a time course in a disease considered to be chronic. The fairly short 12-week duration of this study presents limitations, but the results promote an interesting stratification strategy in that low baseline peripheral blood eosinophil counts may denote ideal candidacy for anti-IgE therapy. A study that is at least two years in duration and that includes multiple endoscopies to assess disease activity will help us to better understand the value of anti-IgE therapy in this patient cohort with low peripheral blood eosinophil counts.

## Supporting Information

S1 Protocol Clinical Trial Protocol. (PDF)

S1 TREND Checklist (PDF)
