# Supplementary material for: A Pilot Study of Omalizumab in Eosinophilic Esophagitis
Source: PLoS One. 2015 Mar 19;10(3):e0113483. doi: 10.1371/journal.pone.0113483 (PMC4366078; doi:10.1371/journal.pone.0113483)

Fig S1. Clinical Study Protocol

Identifying responders to Xolair (omalizumab) using Eosinophilic esophagitis as a disease model.

Author(s):	Oral Alpan, Catherine Chao, Lynn Duffy, Ben Enav, Peter Lee, Ian Leibowitz, Otto Louis-Jacques, Suhasini Kaushal

Phase:	IV
CTRC approval Date	August 6, 2008; protocol Q4513s
Version Dates	January 20, 2009

This Template is a Property of Novartis Pharmaceuticals Corporation and/or Genentech, Inc. Confidential May not be used, divulged, published or otherwise disclosed without the consent of Novartis Pharmaceuticals Corporation and/or Genentech, Inc.

Genentech Inc.
1 DNA Way, South San Francisco, CA 94080-4990

Novartis Pharmaceuticals Corporation
1 Health Plaza, East Hanover, NJ 07936-1080

Protocol synopsis

Title of study: Identifying responders to Omalizumab (Xolair) using eosinophilic esophagitis as a disease model.
Objectives: Our primary objective is to determine markers that will predict responders to Omalizumab (Xolair). Secondary objectives are to look at the immunologic changes in response to therapy.
Study Rationale: Since standard therapy of eosinophilic esophagitis requires obtaining esophageal biopsies, it provides a unique disease model to study allergic inflammation. We have shown (preliminary data) the presence of distinct subgroups based on the presence or absence of certain cells and soluble factors. The rationale for the study is to assess the functional role of anti-IgE therapy in these different sub-groups.
Methodology: We will group patients with eosinophilic esophagitis into 4 different histological subgroups and compare clinical and histological response to treatment with Omalizumab (Xolair).
Number of centers & patients: One center (O & O ALPAN) and a total of 24 patients.
Population: Ages 12-70 with eosinophilic esophagitis and a serum IgE level between 30-700 IU/L.
Investigational drug: Omalizumab (Xolair). Dosage based on weight and serum IgE level.
Reference therapy: Dose will be individualized based on the same dosing chart in all four subgroups. No placebo group.
Study duration: Planned first patient visit September 1, 2008, Last patient last visit August 1, 2009. Total duration of treatment/patient is 3 months.
Evaluation criteria: Clinical and immuno-histological response to Xolair (omalizumab).
Evaluation schedule:

**Visits**	**Screenin g**	**Assignment**	**month 1** (or bi-monthly)	**month 2** (or bi-monthly)	**month 3** (or bi-monthly)
Inclusion/Exclusion criteria	x				
Information & Informed consent	x				
Physical examination	x	x	x	x	x
Upper GI Endoscopy	x				x
Study Drug (bi-weekly or monthly)		x	x	x	
Confocal & light Microscopy	x				x
Adverse events		x	x	x	x
Symptom surveys	x	x	x	x	x
Review of stopping criteria			x	x	x
Laboratory studies (only monthly)		x	x	x	x

Table of contents

Protocol synopsis 2
Ethics and Good Clinical Practice 4
1 Introduction 4
2 Study objectives 7
3 Investigational plan 8
3.1 Overall study design 8
3.2 Study population 9
3.3 Treatments 10
3.4 Visits and Assessments 12
3.5 Efficacy assessment 14
3.6 Safety assessments .... **Error! Bookmark not defined.**17
4 Data management and statistical methods 20
5 References 20
6 Signatures and addresses 22
7 Appendix 23

- **Ethics and Good Clinical Practice**

This study will be performed according to the principles of Good Clinical Practice [Chapter 2 of the ICH Harmonized Tripartite Guideline for Good Clinical Practice (GCP)], the declaration of Helsinki, and national laws and regulations about clinical studies. The study may not start without written Institutional Review Board/Independent Ethics Committee/Research Ethics Board approval and the written informed consent of the patient.

1 Introduction

In various biological systems, certain markers guide to selection of drugs. For example, the estrogen receptor in cancerous breast tissue dictates favorable response to hormone therapy and expression of CD25 on malignant lymphoid cells indicates response to anti-IL-2 therapy in lymphoma (1). At the present time, there are no markers (soluble or cellular) that dictate the use of a specific treatment method in allergic disease. Although IgE levels are taken into account in selecting and dosing for omalizumab, it does not, by itself, indicate favorable response to therapy. Here, we propose to use eosinophilic esophagitis as a model to define

tissue markers that may guide the type of response to omalizumab.

Eosinophilic esophagitis is characterized by the accumulation of eosinophils in the lamina propria and epithelial layer of the distal esophagus. Vomiting, epigastric pain, dysphagia, and heartburn are common presenting symptoms. Patients with eosinophilic esophagitis are predominantly young males and have relatively high numbers of eosinophils (>15 eosinophils/high power field) in the esophageal mucosa, extensive epithelial hyperplasia, a high rate of atopic disease (2).

Although histologically there seems to be a consensus on the criteria for diagnosis, clinically eosinophilic esophagitis is a very heterogeneous disease. First, even though food allergies are implicated to be the cause, only about 40% of patients have detectable food sensitivity on skin testing (prick and patch testing) and only a fraction of them improve with avoidance. Second, 10-20% of patients have no detectible evidence for food or environmental allergies. Third, and most importantly, the response to therapy is variable. Failure to respond to food avoidance and/or steroids is common (2, 3 and our own experience).

At least two different mechanisms can lead to eosinophil accumulation in the esophagus. The first one is the conventional hypothesis, which suggests the formation of Th2 cells upon encounter with allergens (2). These Th2 cells produce IL-4 and IL-13, which influence intestinal epithelial cells to produce eotaxin, a chemokine that attracts eosinophils. Our hypothesis (here termed as the alternative model) suggests that in a subgroup of patients, IL-5 is made predominantly from a non-T cell source (e.g. mast cells). Immunosuppressant medications, such as steroids, may not be as effective in these patients, whereas blocking IgE could be a better approach to therapy. A recent study has shown Xolair (omalizumab) to induce disease remission in approximately 30% of patients with eosinophilic gastrointestinal disease. Although this study did not look at cytokines in the tissue, we believe the responders had significant IL-5 production from mast cells.

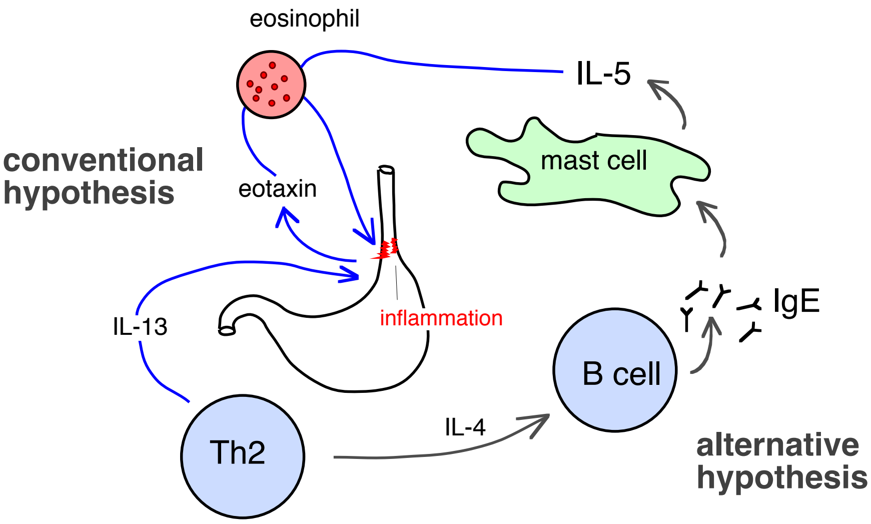

Preliminary observations:

Since response to different treatment modalities are variable and the sensitivity of food testing is limited, we chose to further investigate the pathology in patients with eosinophilic esophagitis using markers that detect the presence of an allergic reaction. To demonstrate the presence of possible different subgroups of patients with eosinophilic esophagitis, we chose to stain esophageal biopsy specimens of patients with antibodies against tryptase, IL-5 and IgE. We then used secondary antibodies tagged with different fluoro-chromes and examined the tissues under confocal microscopy. We chose this technique since it allows 1) three dimensional viewing of the tissue and 2) allow co-localization studies, e.g. asking whether IL-5 and IgE is expressed in the same cell that stains positive for tryptase. This is not possible to do under a light or fluorescent microscope, due to the limitations of 2 dimensional imaging.

These pathology stainings show four different sub-groups of patients with eosinophilic esophagitis. They are summarized in the table below. The first column shows the source of IL-5. The next 4 columns show demographic and laboratory features such as age, IgE level and the presence of food and pollen allergy. The last two columns show response to therapy. It is interesting to see that the most significant response to food avoidance is in the group where IgE and IL-5 expression is linked to mast cells. We believe these patients have truly IgE mediated disease and would most likely benefit from therapy aimed towards IgE itself (see appendix 3).

IL-5	Age (+/-)	IgE (+/-)	Allergies Food	Allergies Pollen	Response to therapy avoidance*	Response to therapy steroid**
Mast cell	12 (7)	326 (134)	8/14	13/14	4/8	9/14
co-localize with IgE#:	10 (5)	294 (95)	5/8	4/8	4/5	7/8
T cells***	11 (7)	411 (178)	7/12	12/12	3/7	5/12
No IL-5	9 (7)	132 (110)	3/6	4/6	1/6	2/6

* Avoidance is recommended based on positive skin test and RAST for foods.
** Inhaled steroids to swallow is the standard of care in EoE, even though treatment failure is common.
*** Although we did not stain for T cells, aside from mast cells, the only other cells reported to make IL-5 are lymphocytes (predominantly Th2 cells).
\# This is the group we believe will be the best responder to Omalizumab, although we expect a statistically significant difference between the mast cell group in general compared to the other groups.

2 Study objectives

Our primary objective is to determine markers that will predict responders to Omalizumab (Xolair). We will use eosinophilic esophagitis as a disease model and will determine clinical and histological changes (and improvements) in response to treatment with Xolair (omalizumab). A recent study has shown that Omalizumab (Xolair) induces disease remission in a 30% of treated patients (4), and our objective will be to identify markers that will predict this favorable response. A patient will be considered to have complete clinical improvement if they are asymptomatic (determined by the absence of vomiting, heartburn, dysphagia and epigastric pain). A partial treatment success will be a 50% reduction in symptoms or <1 symptom/week. A patient will be considered complete histological improvement if there are 1) less than 5 eosinophils/high power field at 3 months and 2) absence of IL-5 staining by confocal microscopy. A partial treatment success will be a 50% reduction in eosinophils/high power field or 50% reduction of the number of cells expressing IL-5 in high power field.

Secondary objectives will be determining the immunological changes in the tissue before and after treatment with Xolair (omalizumab). We will specifically look at IL-13, made by T cells, which acts on esophageal epithelial cells to induce eotaxin. We will also determine the relative localization of T cells, eosinophils and mast cells and their cytokine (IL-13 and eotaxin) production.

3 Investigational plan

3.1 Overall study design

This is an open label study looking at the efficacy of Xolair in eosinophilic esophagitis.

Inclusion criteria

1) Established diagnosis of eosinophilic esophagitis, determined by eosinophils >15/high power field in the distal esophagus
2) Patients should be on therapy either by food avoidance or swallowed steroids, with no change in the food avoidance and steroid dose during therapy.
3) One active symptom of disease (epigastric pain, vomiting, dysphagia or heartburn) at least 2 days of the week.
4) Failed response to proton pump inhibitors or a negative ph probe test.
5) Males and females between ages 12-70 years.
6) Negative impedence study.

Exclusion criteria

1) Patients with gastrointestinal reflux disease.
2) Eosinophilic disease in the stomach or duodenum.
3) Peripheral eosinophil counts >1500 (hyper eosinophilic syndrome).
4) Women of childbearing potential not using the contraception method(s) specified in this study (specify), as well as women who are breastfeeding.
5) Known sensitivity to study drug(s) or class of study drug(s).
6) Patients with severe medical condition(s) that in the view of the investigator prohibits participation in the study (specify as required).

7) Use of any other investigational agent in the last 30 days.
8) Use of systemic or inhaled steroids within the past 1-month.
9) History of malignancy.
10) Require chronic immunosuppressive therapy including cyclosporine, methotrexate, etc.
11) Have been treated with Xolair within the 12 months prior to screening.
12) Patients with eosinophilic esophagitis in remission on swallowed steroids.
13) Patients with asthma taking inhaled steroids.

3.2 Study population

The study population will have to meet the inclusion criteria and fall into one of the following four groups based on confocal microscopy findings of the distal esophagus (Appendix 3).

Group 1: IL-5/Tryptase co-localizing. IL-5 is only being made by mast cells.

Group 2: IL-5/Tryptase/IgE co-localizing. This is possibly a variant of group 1. This is the group where we expect the most significant response to Omalizumab (Xolair).

Group 3: IL-5/Tryptase not co-localizing. Source of IL-5 is a non-mast cell, possibly a T cells. We will also stain T cells and do co-localization studies with IL-5.

Group 4: Mast cells present but no IL-5 staining. Conventional hypothesis is possibly operational in this group. We expect the least response to Omalizumab (Xolair) from this group.

3.3 Treatments

Investigational therapy and reference therapy

Each group will have 6 patients and all will receive Omalizumab (Xolair). Total number of patients enrolled will be 24. Omalizumab (Xolair) dose will be determined using established guidelines based on patient weight and serum IgE level and subjects will be dosed either bi-weekly or monthly. Treatment duration will be 3 months. The different subgroups will act as reference/control for each other.

Treatment assignment and blinding

Treatment assignment will be decided once the confocal microscopy images are reviewed. Based on the findings in the images, patients will be assigned to one of 4 groups as described under study population. To prevent bias, the sub-investigator responsible from the confocal imaging studies will be blinded to the patient name and clinical history. The blinding will only be broken in an emergency.

The principal investigator, based on co focal microscopy findings, will perform the randomization. The investigator may break the randomization code only in a medical emergency. The reasons for this have to be documented carefully.

Concomitant therapy

All concomitant therapy will be noted in the patient's source documents.

Allowed Mediations;

1) Swallowed steroids (Flovent or Budesonide). As long as the dose is kept stable, and the patient is still symptomatic as described in the second inclusion criteria.
2) Singulair.
3) Nasal steroids or nasal antihistamines.
4) Allergen immunotherapy.
5) Oral antihistamines.

Prohibited medications;

1) Oral steroids (from 1 month prior to the completion of study).
2) Change in the dose of swallowed steroids.
3) All immunosuppressive (e.g. cyclosporine, Tacrolimus, etc…) and immunomodulator (IVIG, etc…) therapies.

Interruption or discontinuation of treatment

Patients with uncontrolled disease and in significant discomfort such as persistent vomiting, abdominal pain, food impaction in the esophagus and diarrhea will be discontinued from the study based on the discretion of the principal investigator.

Every patient has the right to discontinue study participation at any time, and every patient may be discontinued from the study for any reason beneficial to his/her wellbeing. All data generated up to the time of discontinuation from the study will be analyzed and the reason(s) for discontinuation will be recorded.

Treatment compliance

Patients will be responsible from taking their maintenance medications either for the eosinophilic esophigitis or other concomitant allergic disease they may have. The study drug (Xolair [omalizumab]) will be kept at study site and administered by study staff. At each visit, compliance of the maintenance medications will be recorded on the source documents.

3.4 Visits and Assessments

Visit schedule and assessments

Screening:

Patients with established eosinophilic esophagitis will be given an informed consent/assent, evaluated for inclusion/exclusion criteria. Patients will then be given a physical exam and a complete allergy evaluation including skin prick and patch testing. Patients will need to have at least one active symptom of disease (vomiting, heartburn, dysphagia or epigastric pain) and an upper gastrointestinal endoscopy within 3 months of screening. Patients with established disease, who are still symptomatic, will need a repeat endoscopy if they have not had one 3 months prior to screening. Qualify patients will have confocal microscopy of their tissue specimens to determine which study group to be randomized in.

Confocal microscopy will be done within 2 weeks of screening. And patients will be assigned to a group on Day 0.

Physical examination & Allergy evaluation:

Skin prick and intra-dermal testing for food and environmental allergens, will be performed as described elsewhere (5, 6). The results will be measured in mm for the wheal and flare reaction. Skin patch testing will be applied using fresh food items and the test results will be measured at 48 hours (5). We have developed a standard imaging method for documentation patch test results as described below. Environmental allergens to be tested will include tree, grass, weed pollen, molds, cat, dog, cockroach, and dust mites. Foods will include soy, wheat, egg, peanut, milk, beef, chicken, pork, oat, rice, barley, broccoli, corn, carrot, potato, apple, orange, grape, peach, banana, yeast, seafood and any other food that is reported by the patient or a caretaker as one that causes symptoms.

Photographic imaging of patch test results:

Documentation of patch test results will be performed by digitally imaging approximately 50cm^2 area of patient's skin per applied test patch. Potentially, patch test results will be collected form patients that are located in diverse geographic locations throughout the region. This fact led us to develop a self-contained digital imaging kit that could be easily used by the patient to perform the imaging at home. The digital imaging kit is designed to yield accurate and consistent results and does not require a particular skill to operate. The kit consists of a fixed-focus digital camera fitted with a close-up lens and stand-off/alignment brackets used together with a disposable self-adhesive (Scanpor Hypo-Allergenic Paper Tape) label that the patient would temporarily apply on the surrounding skin area of the actual patch test. The above-mentioned label will have patient information, as well as millimeter reference scale and alignment marks printed on it.

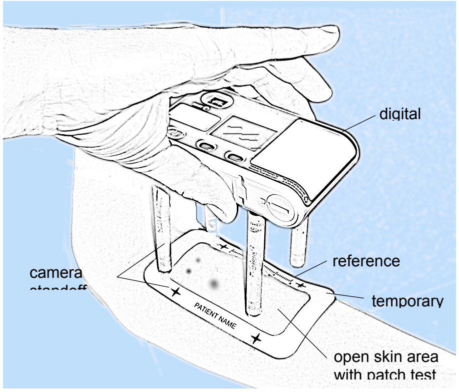

Confocal Microscopy

Confocal microscopy will be performed after the screening, within 2 weeks before randomization. The confocal microscopy will be done at the study site. Tissue samples to be processed for the microscopy will be obtained from the pathology department at Inova Fairfax hospital, where all the endoscopies will be performed. The samples (paraffin blocks) will be transported to our site by either the principal or the sub-investigator).

Immunofluorescent staining

Immunofluorescence studies will be carried out on 7-10 um paraffin-embedded, slide mounted tissue sections from esophagus biopsy samples obtained from affected area. Formalin fixed tissues were de-parafinized in xylene for 9 minutes and underwent serial ethanol dehydration, and. 4% formic acid was applied for 3 minutes for antigen retrieval. All tissue sections were treated with 0.3 % Triton-X 100 (*v/v*) (Sigma) and 0.1 % BSA (*wt/v*)(MPI Biochemicals, Solon, Ohio) to block nonspecific reactions. Immunofluorescence staining will be performed using rabbit anti-human tryptase (1:400), goat anti-human IgE (1:400), mouse anti-human IL- 5 (1:200), rat anti-human IL-13, rabbit anti-human eotaxin, goat anti-human CD3 and mouse anti-human PRG2 monoclonal antibodies overnight at 4°C. FITC, CY3 and AMCA conjugated secondary antibodies will be applied for one hour, and

immunofluorescence detection will be performed on a scanning 510 NLO Meta scanning confocal microscope (Carl Zeiss GmbH, Heidelberg, Germany). We will specifically look at tryptase/IL-5/IgE (panel I), IL-13/IL-5/eotaxin (Panel II) and CD3/PRG2/tryptase (Panel III) combinations. During confocal microscopy a multitrack channel will used to prevent bleed-through, and pinholes will be set at 1.12 Airy units (AU), which corresponds to an optical slice of 0.8µm for both channels. All confocal datasets will be of frame size 512 pixels by 512 pixels, scan zoom of 1 and line averaged 2 or 4 times. All images will be processed using the Zeiss AIM software version 4.0 sp1 (Carl Zeiss GmbH, Heidelberg, Germany).

Symptoms survey cards:

Patients will be provided symptom survey cards to rate their symptoms (see Appendix 1 and 2). Patients will fill out a symptom scale card weekly.

Safety labs:

All patients will have safety labs (CBC and differential, basic chemistry panel, urinalysis and an immune panel at screening, assignment, and at the monthly visits).

Subject Assignment and subsequent visits:

Patients will be assigned into one of four different subgroups based on the findings in confocal microscopy. The first dose of Xolair (omalizumab) will be given at the assignment visit along with survey cards to fill out on a weekly basis. These diaries will be review at each subsequent monthly visit. Based on serum IgE levels and weight, dosing will either be bi-weekly or monthly. There will not be study drug dosing at the last visit, and an upper gastrointestinal endoscopy will be scheduled within one month along with a repeat light microscopy and confocal microscopy to assess response to Omalizumab (Xolair). Patients may require either bi monthly or monthly visits, based on the omalizumab dosing, which depends on the patients weight and serum IgE level.

3.5 Efficacy assessment

The primary dependent measures for the study will be symptom reduction, histological improvement (determined by reduction of tissue eosinophilia) and decrease in the number of IL-5 staining cells in the IL-5/Tryptase/IgE co-localizing group (Group 2). We do not expect

any change in the IL-5 staining in Group 1 or 3. Group 4 will provide an internal control since in the absence of IL-5 and IgE staining we would not expect this group to be a responder. A patient will be considered to have complete symptomatic treatment success if they are asymptomatic. A partial treatment success will be a 50% reduction in symptoms or <1 symptom/week. A patient will be considered a complete histological success if there are <5 eosinophils/high power field, or >70% decrease in the number of cells staining positive for IL-5 at 3 months. Partial histological success will be defined as any number of eosinophils or IL-5 staining cells that fall between 5-15/high-power field and 20-70% decrease respectively.

Secondary efficacy points will be reductions in other eosinophil chemotactic factors not made by mast cells such as IL-13 and eotaxin. We hypothesize that we will not see a major effect on the expression of these cytokines, unless Omalizumab (Xolair) treatment has indirect effects on T cells (for IL-13) or tissue cells (for eotaxin). The staining tissue specimens as outlined under laboratory test will make these assessments.

Specifically, the following staining panels will be done before and after treatment. We will compare staining intensity, number of cells stained and the relative localization of cells/cytokines within the distal esophageal tissue.

Panel I: Trytptase (rabbit) / IL-5 (mouse) / IgE (goat)

This will be the primary endpoint staining. The species in which the antibodies are raised is shown in parenthesis. The secondary staining for confocal microscopy will be done using fluorescent antibodies red, green and blue respectively against rabbit mouse and goat.

The following panels will also be done before and after therapy and will serve to better understand disease mechanism and response to therapy.

Panel II: IL-13 (rat) / IL-5 (mouse) / Eotaxin 1 (rabbit)

IL-13, made by T cells, is thought to act on esophageal epithelial cells to induce eotaxin 1, which is a strong chemotactic factor for eosinophils. We believe, this mechanism may play a significant role in our patients that fall into Groups 3 and 4. These are groups we believe will have the least or no response to Omalizumab (Xolair).

Panel III: CD3 (goat) / PRG2 (mouse) / Tryptase (rabbit)

CD3 stains for T cells, PRG2 for eosinophil major basic protein, and tryptase for mast cells. This panel will give us an idea on the relative localization of each cell population in different groups of patients both pre and post treatment.

3.6 Safety assessments

Definitions

Adverse event

The term adverse event covers any sign, symptom, syndrome, or illness that appears or worsens in a subject during the period of observation in the clinical study and that may impair the well being of the subject. The term also covers laboratory findings or results of other diagnostic procedures that are considered to be clinically relevant (e.g., that require unscheduled diagnostic procedures or treatment measures, or result in withdrawal from the study).

The adverse event may be:

- Worsening of a sign or symptom of the condition under treatment beyond what is typically experienced by the subject
- Worsening of a sign or symptom of a concomitant illness
- An effect of the study medication
- A new illness
- A combination of two or more of these factors.

No causal relationship with the study medication or with the clinical study itself is implied by the use of the term "adverse event".

Surgical procedures themselves are not adverse events; they are therapeutic measures for conditions that require surgery. The condition for which the surgery is required may be an adverse event. Planned surgical measures permitted by the clinical study protocol and the condition(s) leading to these measures are not adverse events.

Adverse events fall into the categories "non-serious" and "serious".

Serious adverse event

A serious adverse event is one that at any dose:

- Results in death
- Is life-threatening[1]
- Requires subject hospitalization or prolongation of existing hospitalization (excluding short hospital stays for study or GI disease required procedures, eg, endoscopy)
- Results in persistent or significant disability/incapacity[2]
- Is a congenital anomaly/birth defect
- Is medically important[3]

[1] "Life-threatening" means that the subject was at immediate risk of death at the time of the serious adverse event; it does not refer to a serious adverse event that hypothetically might have caused death if it were more severe.
[2] "Persistent or significant disability or incapacity" means that there is a substantial disruption of a person's ability to carry out normal life functions.
[3] Medical and scientific judgment should be exercised in deciding whether other adverse events may be considered serious because they jeopardize the subject or may require intervention to prevent one of the other outcomes listed in the definition above. Examples of such events are intensive treatment in an emergency room or at home for allergic bronchospasm, blood dyscrasias or convulsions that do not result in hospitalization, and development of drug dependency or drug abuse.

Cases involving cancer as an adverse event should be reported as "serious" using the criterion" medically important" if no other serious criterion is met.

Clarification of the difference in meaning between "severe" and "serious"
The term "severe" is often used to describe the intensity (severity) of a specific event (as in mild, moderate, or severe myocardial infarction); the event itself, however, may be of relatively minor medical significance (such as severe headache). This is not the same as "serious", which is based on the outcome or action criteria usually associated with events that pose a threat to life or functioning. Seriousness (not severity) serves as a guide for defining regulatory reporting obligations.

Intensity
The severity of events reported will be determined by the principal investigator as:

Mild: Minor or annoying discomfort with no limitation in normal daily activity
Moderate: Definite discomfort with minimal limitation in normal daily activity
Severe: Interferes with normal daily activity or interrupts sleep

Relationship to study drug

The relationship of treatment to adverse events will be determined by the investigator based on the following definitions:

1. Not related:
   The subject was not exposed to vaccine, or another cause is obvious.
2. Probably Not Related:
   The adverse event is most likely explained by another cause, and the time of occurrence of the event is not reasonably related to vaccine administration.
3. Possibly related:
   Vaccine administration and event occurrence reasonably related in time, and the event is explained equally well by causes other than study vaccine; or vaccine administration and event occurrence are not reasonably related in time, but the event is not obviously a result of other causes.
4. Probably Related:
   Vaccine administration and event occurrence are reasonably related in time, and the event is more likely explained by vaccine exposure than by other mechanisms.

Documentation and reporting of adverse events by investigator

All adverse events (including one specific to the gastrointestinal tract; vomiting, food impaction and diarrhea) that occur after the subject has signed the informed consent document must be documented. If the adverse event is serious, the investigator must complete a "SAE/ER from a Clinical Trial" form at the time the serious adverse event is detected. Every attempt should be made to describe the adverse event in terms of a diagnosis. If appropriate, component symptoms should also be listed below the diagnosis on the SAE/ER from a Clinical Trial form. If only non-specific signs or symptoms are present, then these should be recorded separately as diagnoses. All subjects who have adverse events, whether considered associated with the use of the study medication or not, must be monitored to determine the outcome. The clinical course of the adverse event will be followed up according to accepted standards of medical practice, even after the end of the study, until a satisfactory explanation is found or the investigator considers it medically justifiable to terminate follow-up. Should the adverse event result in death, a full pathologist' s report should be supplied, if possible.

Safety data

Safety will be assessed on the basis of adverse events, clinical laboratory evaluations, physical examinations, and vital signs.

Withdrawals

Subjects may be withdrawn from study medication for the following reasons:

- At their own request or at the request of their legally authorized representative
- If, in the investigator's opinion, continuation in the study would be detrimental to the subject's well-being at the specific request of the sponsor.

Subjects must be withdrawn from study medication under the following circumstances:

- If the study blind has been broken
- If the subject develops a serious interfering medical condition(e.g. cancer), that in the opinion of the investigator, will markedly affect the patient's health/urticaria
- Pregnancy
- Thrombocytopenia
- Increase in the symptoms of eosinophilic esophagitis.

In all cases, the reason for withdrawal must be recorded. The subject must be followed up to establish whether the reason was an adverse event.

Safety assessments will consist of monitoring and recording all adverse events, including serious adverse events, as described in the investigators brochure.

An adverse event is any undesirable sign, symptom or medical condition occurring after starting study drug (or therapy). Medical conditions/diseases present before starting study treatment are only considered adverse events if they worsen after starting study treatment.

A serious adverse event is an undesirable sign, symptom or medical condition which: 1. Are fatal or life-threatening, 2. required or prolonged hospitalization, 3. results in persistent or significant disability/incapacity, 4. constitutes a congenital anomaly or a birth defect, 5. is medically significant, in that it may jeopardize the subject and may require medical or surgical intervention to prevent one of the outcomes listed above.

Individual Patient stopping Criteria

In addition to he abovementioned general stopping criteria, patients will be discontinued from the study due to omalizumab specific adverse events which includes, but not limited to thrombocytopenia, anaphylaxis, allergic reactions, malignancy and serum sickness.

4 Data management and statistical methods

Investigators will enter the information required by the protocol into the Case Report Forms (CRFs). Non-obvious errors or omissions will be entered on Data Query Forms, which will be returned to the investigational site for resolution.

The objective of this trial is to study the efficacy and tolerability of Xolair (omalizumab) in eosinophilic esophagitis. The data will be summarized with respect to demographic and baseline characteristics, subgroups and efficacy and safety observations. For the purpose of sample size estimation, we are hypothesizing a symptomatic and histologic success rate in Group 1, 3 and 4 to be 25-35% versus 75% in Group 2. For a 2-tailed test of $p=0.05$ and a power of 90%, 6 patients per group (N=24) will be required. Repeated measures analysis-of-variance will be used to assess change over time in self-reported symptoms within and between treatment groups. This technique also will be employed to examine histological change over time within and between treatment groups. Binomial success rates for both symptom reduction and histological improvement will be assessed using Fisher's Exact test and odds ratios with 95% confidence intervals. All analyses will be performed using SAS software (v9.1, SAS Institute). A value of $p \leq 0.05$ will be considered as significant.

The assessment of safety will be based mainly on the frequency of adverse events, which includes all serious adverse events. Adverse events will be summarized by presenting for each treatment group the number and percentage of patients having any adverse event, having an adverse event in each body system and having each individual adverse event. Any other information collected (e.g. severity or relatedness to study medication) will be listed as appropriate.

5. References

1. Waldmann TA. Anti-IL-2 receptor monoclonal antibody (anti-Tac) treatment of T-cell lymphoma. *Important Adv Oncol.* 1994; 18: 131-41.
2. Blanchard C. et al., Eosinophilic Esophagitis; Pathogenesis, genetics and therapy. *J Allergy Clin Immunol.* 2006; 118: 1054-63.
3. Konikoff MR. A randomized, double blind, placebo-controlled trial of fluticasone propionate for pediatric eosinophilic esophagitis. *Gastroenterology.* 2006; 131: 1381-91.
4. Foroughi S. et al., Anti-IgE treatment of eosinophil associated gastrointestinal disorders. *J Allergy Clin Immunol.* 2007; 120: 594-601.

5. Oppenheimer J. Skin testing. *Ann Allergy Asthma Immunol.* 2006; 96: 6-12.
6. Spergel JM. Eosinophilic esophagitis in adults and children: evidence for a food allergy component in many patients. *Curr Opin Allergy Clin Immunol*. 2007; 12: 274-278.

6 Signatures and addresses

Signature of Investigator(s) and Study Personnel

Principal Investigator ______________________________ __________
Oral Alpan, M.D. 1/20/09

Head of investigational site ______________________________ __________
Oral Alpan, M.D. 1/20/09

type/print name ______________________________ __________
USMA Medical Director signature date

type/print name ______________________________ __________
(Regional Scientific Director) signature date

Appendix 1

Symptom Survey Card

	Mild = 1	2	3	4	Severe = 5
Vomiting					
Heartburn					
Nausea					
Dysphagia					
Pain					

	Number of Episodes This Week
Vomiting	
Heartburn	
Nausea	
Dysphagia	
Epigastric pain	

Appendix 2

Histology Scoring

	Grade 1<br>15-30	Grade 2<br>30-40	Grade 3<br>40-50	Grade 4<br>>50
# Eosinophils/hpf				
	Comment on the degree			
Basilar hyperplasia				
Vascular papillae elongation				

The color codes for Figures 2-5 are:
Red: Tryptase (Mast cells)
Green: IL-5
Blue: IgE
Co-localization:
Red+Green = Yellow
Red+Green+Blue = White

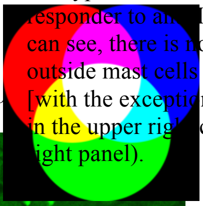

In this tissue specimen, the staining is very similar to the one in Figure 1, however, we can now see IgE staining and co-localization of it to IL-5 producing mast cells (very pale yellow looking cells in the co-localization study). This is the group, we hypothesize, would be the best responder to anti-IgE therapy. As you can see, there is no IL-5 production outside mast cells (no green staining [with the exception of one small area in the upper right corner] in the lower right panel).

As you can see in the right lower panel IL-5 is only made by mast cells, but there is no significant IgE staining.

Appendix 3

Group 1. Mast cells staining [illegible]

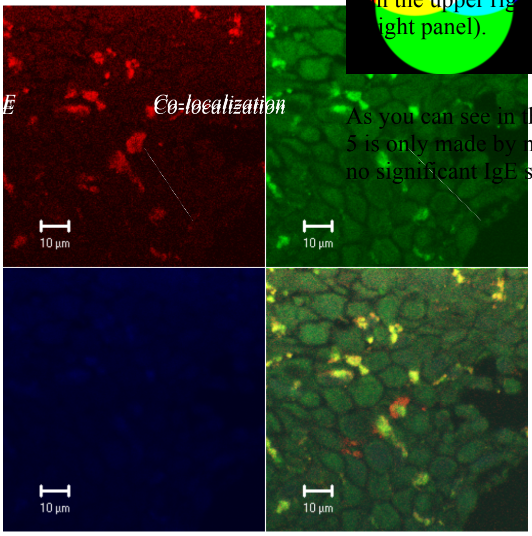

Group 2. Mast cells staining for IL-5 and IgE.

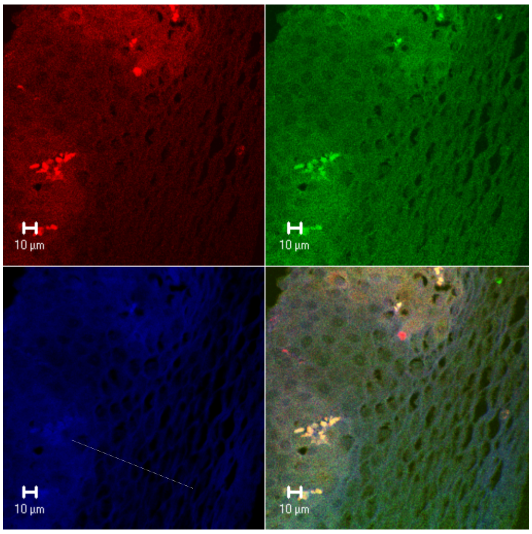

In this tissue specimen, mast cells and IL-5 can be stained, but they do not co-localize, as can be seen in the lower right panel (circles showing IL-5 in green apart from the mast cells staining red). Many of these patients have significant atopy based on history and allergy testing, but the mechanism of EoE is possibly non-mast cell and non-IgE medicated.

In this tissue specimen there are significantly higher numbers of mast cells, but no IL-5 staining. The mechanism of EoE in this patient group is perhaps very different (e.g. eotaxin made by esophageal epithelial cells or IL-13 made by T cells). Note the lack of IgE staining.

Group 3. IL-5 staining outside mast cells.

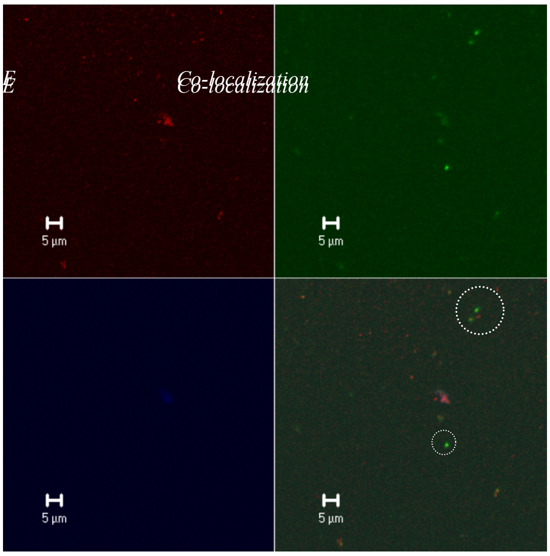

Group 4. Increased mast cells, but no IL-5 staining in tissue.

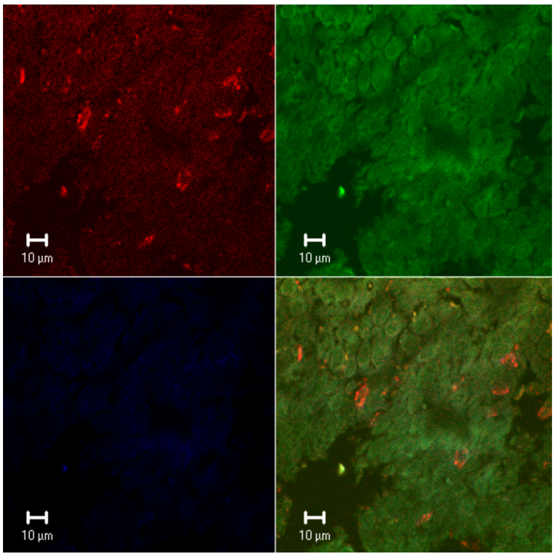

Supplement: S1 Protocol — (PDF) [file pone.0113483.s001.pdf]
